# Supplementary material for: From sequence to enzyme mechanism using multi-label machine learning
Source: BMC Bioinformatics. 2014 May 19;15:150. doi: 10.1186/1471-2105-15-150 (PMC4229970; doi:10.1186/1471-2105-15-150)
Supplement: Additional file 2 — Java code of ml2db. Additional file ml2db_code.tar.gz contains the Java source code to run the multi-label machine learning experiments and save the results to database. The code’s Javadoc is included. [file 1471-2105-15-150-S2.zip › additional file 2/ml2db/ml2db/doc/index-files/index-13.html]

M-Index


JavaScript is disabled on your browser.


- Overview
- Package
- Class
- Use
- Tree
- Deprecated
- Index
- Help

- Prev Letter
- Next Letter

- Frames
- No Frames

- All Classes

A B C D E F G H I J K L M N O P Q R S T U V W X 


## M

m\_arff - Static variable in class test.ml2db.weka.DataSetManagerTest


m\_debug - Static variable in class main.ml2db.al.utility.confidence.ConfidenceMetric


m\_debug - Variable in class main.ml2db.al.utility.pickers.RandomPicker


m\_debug - Static variable in class main.ml2db.mulan.learn.MulanLearners


m\_debug - Variable in class main.ml2db.mulan.mulanarff.MulanInstancesFiller


m\_debug - Static variable in class main.ml2db.mulan.predict.PredictionsRecordTable


m\_distances - Variable in class main.ml2db.al.utility.globaldistance.DistancesStorage
:   Stores the two instances as a string (instance 1 | instance 2)

m\_massiveFile - Variable in class main.ml2db.mulan.mulanarff.MulanArff
:   if plenty of memory available, also load each instance in weka instances,
    otherwise only write them to file one by one

m\_test - Variable in class main.ml2db.al.utility.pickers.RandomPicker


m\_uncertainties - Variable in class main.ml2db.al.utility.confidence.ConfidencesStorage
:   Stores for a set of confidences, its uncertainty (distance between
    average confidence over all labels and 50%)

MACHINE\_LEARNING\_LOCAL\_DB\_PROPS - Static variable in class main.ml2db.ProjectParameters


MACHINE\_LEARNING\_PROPS - Static variable in class main.ml2db.ProjectParameters


MACHINE\_LEARNING\_REMOTE\_DB\_PROPS - Static variable in class main.ml2db.ProjectParameters


MACIE\_HOM\_DB\_PROPS - Static variable in class main.ml2db.ProjectParameters


main(String[]) - Static method in class main.ml2db.al.ActiveLearning


main(String[]) - Static method in class main.ml2db.al.utility.globaldistance.DistancesCalculator


main(String[]) - Static method in class main.ml2db.al.utility.globaldistance.GlobalDistanceMetric


main(String[]) - Static method in class main.ml2db.al.utility.neighbours.NeighbourhoodExperimentDetailsSerialised


main(String[]) - Static method in class main.ml2db.al.utility.neighbours.NeighbourhoodNetwork


main(String[]) - Static method in class main.ml2db.DbManagers


main(String[]) - Static method in class main.ml2db.graphviz.GraphVizPrinter


main(String[]) - Static method in class main.ml2db.mulan.attributesfiltered.AttFilter


main(String[]) - Static method in class main.ml2db.mulan.learn.crosseval.CrossExperimenter


main(String[]) - Static method in class main.ml2db.mulan.learn.crosseval.EvaluationResultsTable


main(String[]) - Static method in class main.ml2db.mulan.learn.MulanLearners


main(String[]) - Static method in class main.ml2db.mulan.learn.MulanSerializer


main(String[]) - Static method in class main.ml2db.mulan.learn.traintest.TrainTestExperimenter


main(String[]) - Static method in class main.ml2db.mulan.learn.traintest.TrainTestExperimenterSerialized


main(String[]) - Static method in class main.ml2db.mulan.learn.traintest.TrainTestFullRun


main(String[]) - Static method in class main.ml2db.mulan.measuremetrics.PredictionsPerformanceMeasurer


main(String[]) - Static method in class main.ml2db.mulan.mulanarff.database.MulanArffRecord


main(String[]) - Static method in class main.ml2db.mulan.mulanarff.ImportMulanArff
:   IMPORTANT: Please note that the arff to be imported should have, before each instance line,
    a line starting with % containing a unique instance identifier.

main(String[]) - Static method in class main.ml2db.mulan.mulanarff.MulanArff


main(String[]) - Static method in class main.ml2db.mulan.mulanarff.NeighboursPrinter


main(String[]) - Static method in class main.ml2db.mulan.predict.QuickPredictor
:   To run a prediction experiment, please provide:
    Input: 1.

main(String[]) - Static method in class main.ml2db.Serializer


main(String[]) - Static method in class main.ml2db.weka.Arff


main(String[]) - Static method in class main.ml2db.weka.ImportArff
:   IMPORTANT: Please note that the arff to be imported should have, before each instance line,
    a line starting with % containing a unique instance identifier.

main(String[]) - Static method in class main.ml2db.weka.JaccardDistanceStored


main(String[]) - Static method in class test.ml2db.alltests.AllActiveLearningTests


main(String[]) - Static method in class test.ml2db.alltests.AllArffTests


main(String[]) - Static method in class test.ml2db.alltests.AllDataTests


main(String[]) - Static method in class test.ml2db.alltests.AllExternalPredictionsEvaluationTests


main(String[]) - Static method in class test.ml2db.alltests.AllGraphVizTests


main(String[]) - Static method in class test.ml2db.alltests.AllMulanArffTests


main(String[]) - Static method in class test.ml2db.alltests.AllMulanLearningTests


main(String[]) - Static method in class test.ml2db.alltests.AllMulanPredictionTests


main(String[]) - Static method in class test.ml2db.alltests.AllMulanTests


main(String[]) - Static method in class test.ml2db.alltests.AllPreliminaryTests


main(String[]) - Static method in class test.ml2db.alltests.AllTests


main(String[]) - Static method in class test.ml2db.alltests.AllTrainTestsTests


main(String[]) - Static method in class test.ml2db.mulan.predict.MulanEmitPredictions


main.ml2db - package main.ml2db


main.ml2db.al - package main.ml2db.al


main.ml2db.al.database - package main.ml2db.al.database


main.ml2db.al.learning - package main.ml2db.al.learning


main.ml2db.al.utility - package main.ml2db.al.utility


main.ml2db.al.utility.confidence - package main.ml2db.al.utility.confidence


main.ml2db.al.utility.globaldistance - package main.ml2db.al.utility.globaldistance


main.ml2db.al.utility.hybrid - package main.ml2db.al.utility.hybrid


main.ml2db.al.utility.neighbours - package main.ml2db.al.utility.neighbours


main.ml2db.al.utility.pickers - package main.ml2db.al.utility.pickers


main.ml2db.al.utility.pickers.byfrequency - package main.ml2db.al.utility.pickers.byfrequency


main.ml2db.graphviz - package main.ml2db.graphviz


main.ml2db.mulan.attributesfiltered - package main.ml2db.mulan.attributesfiltered


main.ml2db.mulan.classifier.lazy - package main.ml2db.mulan.classifier.lazy


main.ml2db.mulan.learn - package main.ml2db.mulan.learn


main.ml2db.mulan.learn.crosseval - package main.ml2db.mulan.learn.crosseval


main.ml2db.mulan.learn.evaluation - package main.ml2db.mulan.learn.evaluation


main.ml2db.mulan.learn.traintest - package main.ml2db.mulan.learn.traintest


main.ml2db.mulan.measuremetrics - package main.ml2db.mulan.measuremetrics


main.ml2db.mulan.mulanarff - package main.ml2db.mulan.mulanarff


main.ml2db.mulan.mulanarff.database - package main.ml2db.mulan.mulanarff.database


main.ml2db.mulan.predict - package main.ml2db.mulan.predict


main.ml2db.weka - package main.ml2db.weka


makeInstances() - Method in class main.ml2db.weka.DataSetGenerator
:   an array of zeros as long as the number of attributes, to reset all
    nominal (binary) attribute values of an instance to 0 (false,absent)

manyFilterRuns() - Static method in class main.ml2db.mulan.attributesfiltered.AttFilter
:   Executes arff generation and filtering of several train-test files
    couples

MicroFMeasurePrinter - Class in main.ml2db.mulan.learn.evaluation
:   A micro f measure version that also prints out the number of true positives,
    false positives and false negatives

MicroFMeasurePrinter(int) - Constructor for class main.ml2db.mulan.learn.evaluation.MicroFMeasurePrinter


ML\_PROPS\_PATH - Static variable in class test.ml2db.dataharness.TestProjectParameters


MODEL\_FOLDER - Static variable in class test.ml2db.dataharness.TestProjectParameters


MODELS\_PATH - Static variable in class main.ml2db.ProjectParameters


MODELS\_PATH - Static variable in class test.ml2db.dataharness.TestProjectParameters


MulanArff - Class in main.ml2db.mulan.mulanarff
:   Generates an Attribute Relation Format File (ARFF) in Mulan format (multiple
    class labels per instance).

MulanArff(String, DbManagers) - Constructor for class main.ml2db.mulan.mulanarff.MulanArff


MulanArff(String, DbManagers, boolean) - Constructor for class main.ml2db.mulan.mulanarff.MulanArff


MulanArffInstancesLoader - Class in main.ml2db.mulan.mulanarff.database
:   Loads instances from file, given an arff database record identifier.

MulanArffInstancesLoader() - Constructor for class main.ml2db.mulan.mulanarff.database.MulanArffInstancesLoader


MulanArffProperties - Class in main.ml2db.mulan.mulanarff
:   Class

MulanArffProperties(String) - Constructor for class main.ml2db.mulan.mulanarff.MulanArffProperties


MulanArffRecord - Class in main.ml2db.mulan.mulanarff.database
:   Checks that an ARFF record read from the database contains valid parameters
    to read an ARFF file.

MulanArffRecord(int, DbManagers) - Constructor for class main.ml2db.mulan.mulanarff.database.MulanArffRecord


MulanArffRecordTest - Class in test.ml2db.mulan.mulanarff.database
:   Class

MulanArffRecordTest() - Constructor for class test.ml2db.mulan.mulanarff.database.MulanArffRecordTest


MulanArffTest - Class in test.ml2db.mulan.mulanarff
:   Class

MulanArffTest() - Constructor for class test.ml2db.mulan.mulanarff.MulanArffTest


MulanAttributeFactory - Class in main.ml2db.mulan.mulanarff
:   In the Mulan ARFF, the class attribute is not generated.

MulanAttributeFactory(MulanDataSetGenerator) - Constructor for class main.ml2db.mulan.mulanarff.MulanAttributeFactory


MulanDataSetDbLoader - Class in main.ml2db.mulan.mulanarff
:   Loads data from database to a Mulan data factory

MulanDataSetDbLoader(MulanDataSetManager) - Constructor for class main.ml2db.mulan.mulanarff.MulanDataSetDbLoader


MulanDataSetDbLoaderTest - Class in test.ml2db.mulan.mulanarff
:   Class

MulanDataSetDbLoaderTest() - Constructor for class test.ml2db.mulan.mulanarff.MulanDataSetDbLoaderTest


MulanDataSetGenerator - Class in main.ml2db.mulan.mulanarff
:   Class

MulanDataSetGenerator(MulanDataSetManager, InstAttValueMap, InstAttValueMap) - Constructor for class main.ml2db.mulan.mulanarff.MulanDataSetGenerator


MulanDataSetGeneratorTest - Class in test.ml2db.mulan.mulanarff
:   Class

MulanDataSetGeneratorTest() - Constructor for class test.ml2db.mulan.mulanarff.MulanDataSetGeneratorTest


MulanDataSetManager - Class in main.ml2db.mulan.mulanarff
:   This class generates a data set (instances, attributes, classes) in Mulan
    style (for multi-label machine learning).

MulanDataSetManager(MulanArff) - Constructor for class main.ml2db.mulan.mulanarff.MulanDataSetManager


MulanDataSetManagerTest - Class in test.ml2db.mulan.mulanarff
:   Class

MulanDataSetManagerTest() - Constructor for class test.ml2db.mulan.mulanarff.MulanDataSetManagerTest


MulanDataSetWriterTest - Class in test.ml2db.mulan.mulanarff
:   Class

MulanDataSetWriterTest() - Constructor for class test.ml2db.mulan.mulanarff.MulanDataSetWriterTest


MulanDbWriterTest - Class in test.ml2db.mulan.learn.database
:   Class

MulanDbWriterTest() - Constructor for class test.ml2db.mulan.learn.database.MulanDbWriterTest


MulanEmitPredictions - Class in test.ml2db.mulan.predict
:   Class

MulanEmitPredictions() - Constructor for class test.ml2db.mulan.predict.MulanEmitPredictions


MulanInstancesFiller - Class in main.ml2db.mulan.mulanarff
:   Class

MulanInstancesFiller(MulanDataSetGenerator) - Constructor for class main.ml2db.mulan.mulanarff.MulanInstancesFiller


MulanInstancesFillerTest - Class in test.ml2db.mulan.mulanarff
:   Class

MulanInstancesFillerTest() - Constructor for class test.ml2db.mulan.mulanarff.MulanInstancesFillerTest


MulanLearner - Class in main.ml2db.mulan.learn
:   Containing a Mulan learner and its name, that is: the name of the
    MultiLabelLearnerBase plus the name of the algorithm within

MulanLearner(String, String) - Constructor for class main.ml2db.mulan.learn.MulanLearner
:   Incomplete constructor: it is essential to then set a learner of the same
    type specified!

MulanLearner(String, String, MultiLabelLearnerBase) - Constructor for class main.ml2db.mulan.learn.MulanLearner


MulanLearners - Class in main.ml2db.mulan.learn
:   Class

MulanLearners() - Constructor for class main.ml2db.mulan.learn.MulanLearners


MulanLearnerTest - Class in test.ml2db.mulan.learn
:   Class

MulanLearnerTest() - Constructor for class test.ml2db.mulan.learn.MulanLearnerTest


MulanSerializer - Class in main.ml2db.mulan.learn
:   Takes an arff file and an algorithm.

MulanSerializer(MulanArffRecord, MulanLearner, String) - Constructor for class main.ml2db.mulan.learn.MulanSerializer


MulanTrainExample - Class in main.ml2db.mulan.learn.traintest
:   Give arff and xml file path, weka algorithm, cross evaluation fold and it
    emits learning results

MulanTrainExample() - Constructor for class main.ml2db.mulan.learn.traintest.MulanTrainExample

A B C D E F G H I J K L M N O P Q R S T U V W X

- Overview
- Package
- Class
- Use
- Tree
- Deprecated
- Index
- Help

- Prev Letter
- Next Letter

- Frames
- No Frames

- All Classes
